# Representation of three-dimensional space in the auditory cortex of the echolocating bat *P*. *discolor*

**DOI:** 10.1371/journal.pone.0182461

**Published:** 2017-08-16

**Authors:** Wolfgang Greiter, Uwe Firzlaff

**Affiliations:** Chair of Zoology, Department of Animal Sciences, TUM School of Life Sciences Weihenstephan, Technical University of Munich, Freising, Germany; Universidad de Salamanca, SPAIN

## Abstract

The auditory cortex is an essential center for sound localization. In echolocating bats, combination sensitive neurons tuned to specific delays between call emission and echo perception represent target distance. In many bats, these neurons are organized as a chronotopically organized map of echo delay. However, it is still unclear to what extend these neurons can process directional information and thereby form a three-dimensional representation of space. We investigated the representation of three-dimensional space in the auditory cortex of *Phyllostomus discolor*. Specifically, we hypothesized that combination sensitive neurons encoding target distance in the AC can also process directional information. We used typical echolocation pulses of *P*. *discolor* combined with simulated echoes from different positions in virtual 3D-space and measured the evoked neuronal responses in the AC of the anesthetized bats. Our results demonstrate that combination sensitive neurons in the AC responded selectively to specific positions in 3-D space. While these neurons were sharply tuned to echo delay and formed a precise target distance map, the neurons’ specificity in azimuth and elevation depended on the presented sound pressure level. Our data further reveal a topographic distribution of best elevation of the combination sensitive neurons along the rostro-caudal axis i.e., neurons in the rostral part of the target distance map representing short delays prefer elevations below the horizon. Due to their spatial directionality and selectivity to specific echo delays representing target distance, combination sensitive cortical neurons are suited to encode three-dimensional spatial information.

## Introduction

The auditory cortex (AC) plays a major role in the localization of sound sources in mammals [1–3]. Bats are an especially interesting model system to study sound localization as they depend on echolocation to navigate in darkness and therefore have a highly specialized auditory system. In addition to sound localization in azimuth and elevation, bats use the time delay between the emission of the echolocation pulse and the perception of reflected echoes to measure the distance to objects. The coding of sound direction and echo delay was intensively studied in different regions of the auditory pathway in bats, including the superior colliculus and the auditory cortex [4–7]. In the superior colliculus, an auditory space representation with a topographic arrangement of sound azimuth was found [6,7]. In the auditory cortex, however, the current knowledge about spatial sound source coding is rather limited and no clear topographic space map could be found so far. The neurons in the primary fields of the auditory cortex are typically arranged tonotopically and can further encode interaural level differences [8–10]. Spatial positions in azimuth and elevation seem to be encoded by the extent of the activated cortex resulting from the overlap of binaural and tonotopic maps [4,11].

In the non-primary fields of the AC, however, studies in several bats revealed chronotopically arranged combination sensitive neurons encoding echo delay [12–14], thereby forming a topographic representation of distance. These neurons, usually responding to high frequency sounds in the echolocation range [9,13], might therefore be especially suited to further encode spatial direction during active echolocation, thereby forming a three-dimensional representation of space. However, very few studies tried to investigate the directional sensitivity of combination sensitive cortical neurons so far. It remains a matter of debate if these combination sensitive neurons can process directional information and encode three-dimensional acoustic space.

Suga et al. [15] investigated the spatial selectivity of cortical combination sensitive neurons in the bat *Pteronotus parnellii* by presenting pairs of short frequency modulated pulses (FM pulses). They found that these combination sensitive neurons were extremely broadly tuned to auditory space in azimuth and elevation (at 10 dB above threshold: width about 70° in azimuth and elevation, at 30 dB above threshold: outside measurable range) and responded most strongly to sounds delivered from directly in front of the bat. They suggested that combination sensitive neurons are not suited to process directional information but that this information is processed in parallel by a separate population of neurons in the cortex. However, it remained unclear where this directional information could be processed in the cortex and how bats are able to combine the directional information of objects in space with the information about the distance to these object.

In contrast to this study on cortical neurons, combination sensitive neurons in the superior colliculus exhibited a higher spatial selectivity in azimuth to presented pairs of FM pulses and it was proposed that these 3D neurons might play a role in combining azimuthal, elevational and distance cues to guide perceptually driven orientation and vocalization responses [5]. However, the selectivity of these neurons to different positions in elevation was not systematically investigated in this study.

Hoffmann et al. [16,17] investigated the spatiotemporal response characteristics of cortical neurons by presenting sequences of echoes in virtual acoustic space and evaluated them using a reverse-correlation technique. They found that neurons with complex temporal response patterns, possibly corresponding to combination sensitive neurons, exhibited a clear spatial selectivity in azimuth and elevation.

However, all studies mentioned above used rather artificial stimuli to investigate the temporal and spatial selectivity of neurons by presenting pairs or sequences of sounds from stationary loudspeakers or earphones without considering for example sonar emission characteristics. The bats’ sonar system, however, is highly directional. Recent studies showed that *P*. *discolor* as well as other bats have a highly dynamical biosonar and are constantly controlling their biosonar field of view by adjusting their emitter aperture [18,19]. The sonar beam aim and directionality serves to filter and separate target and clutter echoes. Therefore, it is crucial to include call emission characteristics; distance and frequency dependent sound damping as well as the ear directionality in such an investigation.

We aimed our study to fill the gap of knowledge on the three-dimensional representation of auditory space in the auditory cortex. We specifically designed this study to use stimuli corresponding to a more natural situation than the studies mentioned above by including pulse emission characteristics of *P*. *discolor*, and distance and frequency dependent sound damping as well as all relevant spatial cues such as interaural time and level differences and spectral differences due to the head related transfer function (HRTF).

Our hypothesis was that combination sensitive neurons encoding target distance in the auditory cortex of *Phyllostomus discolor* can also process directional information. To test this hypothesis, we presented combinations of typical echolocation pulses of *P*. *discolor* and virtual echoes from different simulated positions in 3D-space and measured the evoked neuronal responses of combination sensitive neurons in the posterior dorsal field (PDF) of the AC in anesthetized bats.

We found that neurons tuned to specific echo delays responded selectively to specific spatial positions in 3-D acoustic space. Neurons mainly encoded positions in the frontal and lower contralateral space. Spatial directionality in elevation was related to the encoded delay range. Spatial selectivity increased at lower sound pressure levels.

## Methods

### Animals and surgery

All experiments complied with the principles of laboratory animal care and were conducted under the regulations set out by the current version of the German Law on Animal Protection (approval 55.2-1-54-2532-147-13 Regierung von Oberbayern). Three bats (*Phyllostomus discolor*, all males) originated from a breeding colony situated in the Department Biology II of the Ludwig-Maximilian University of Munich. The animals were kept separated from other bats under semi-natural conditions (reversed 12 h day/12 h night cycle, 65%–70% relative humidity, 28°C) with free access to food and water.

Initial surgery and all following electrophysiological experiments were performed under anesthesia. The bats were anesthetized using a combination of medetomidine (Dorbene^®^, Zoetis), midazolam (Dormicum^®^, Hoffmann-La Roche) and fentanyl (Fentadon^®^, Albrecht) at a dosage of 0.4, 4.0 and 0.04 μg/g body weight, respectively. Anesthesia was maintained for up to 5 h through additional injections containing two-thirds of the initial dose every 1.5 h. After completion of surgery or experiments, the anesthesia was antagonized with a mixture of atipamezole (Alzane^®^, Novartis), flumazenil (Flumazenil, Hexal) and naloxone (Naloxon-ratiopharm^®^, Ratiopharm), which was injected subcutaneously (2.5, 0.5 and 1.2 μg/g body weight, respectively).

The initial surgical procedures are described in detail by Hoffmann et al. [9] and will be mentioned only briefly here. The skin overlying the skull was opened along the midline and the skull surface was freed from tissue. A small metal tube was then fixed to the skull using a microglass composite in order to immobilize the animal in a stereotaxic device during the experiments. Details of the stereotaxic device and the procedure used to reconstruct the recording sites are described elsewhere [20]. In brief, the alignment of the animal’s skull and the underlying brain within the stereotaxic coordinate system was measured by scanning the characteristic profile lines of the skull in the parasagittal and frontal planes. These profiles were then fitted onto a standardized skull profile in a standardized coordinate system. To alleviate postoperative pain, an analgesic (0.2 μg/g body weight; Meloxicam, Metacam, Boehringer-Ingelheim) was administered after the surgery for four postoperative days. In addition to that, the bats were treated with antibiotics (0.5 μg/g body weight; enrofloxacin, Batril^®^, Bayer) for four postoperative days.

### Electrophysiological recordings

After initial surgery, experiments were conducted in a sound-attenuated and heated (~35°C) chamber. Extracellular recordings were made with parylene-coated tungsten microelectrodes (5 MΩ impedance, Alpha Omega) in anesthetized bats (see above). Note that the responses recorded from cortical units under this anesthesia regime reflect the behavioral performance of *P*. *discolor* well [21]. Recording sessions took place three days a week for up to eight weeks (with at least one day off between consecutive experiments) and could last up to five hours per day. Electrode penetrations in the AC in both hemispheres were run obliquely to the brain surface with different mediolateral and rostrocaudal angles. The electrode signal was recorded using an AD converter (RA16, RX5 (Tucker-Davis Technologies; TDT), sampling rate 25 kHz, band-pass filter 400–3000 Hz) and Brainware (TDT). The action potentials were threshold discriminated and saved for later offline analysis. Single neurons were analyzed preferentially. However, as it was not always possible to clearly discriminate the activity of a single neuron, the term “unit” will be used in this paper to describe the collective activity of one to three neurons recorded at a recording site.

In order to search for acoustically driven neural activity, pairs of typical echolocation calls of *P*. *discolor* (representing “pulse” and a virtual “echo”) were used (downward modulated, multiharmonic, main energy between 40 and 90 kHz, duration approximately 1.2 ms). The delay and amplitude ratio between the “pulse” and “echo” could be varied (DA converter: RX6 (TDT), sampling rate 260 kHz, attenuator: PA5 (TDT)). Search stimuli were presented diotically (without binaural time, level or spectral differences) with a repetition rate of 2 Hz. The use of search stimuli without ILDs or spectral differences might introduce a small bias to midline selective neurons. However, our results demonstrate changing spatial selectivity of the recorded neurons and an overall tendency to respond to stimuli in the contralateral hemisphere. For units that responded to these presented pairs of search stimuli, basic delay response properties (without any spatial cues) and the responses to simulated pulses and echoes from different spatial positions (including all relevant spatial cues) were determined (see the following section). All acoustic stimuli were presented *via* custom-made transducer ear phones (flat frequency response of ±10 dB between 10 kHz and 120 kHz, [22]). The ear phones were calibrated using a Brüel & Kjaer reference microphone (type 4939) and a Brüel & Kjaer Measuring Amplifier (type 2610).

After completion of all electrophysiological experiments, a neuronal marker (BDA 3000, Sigma-Aldrich, 1 mg/20 μl phosphate buffer) was pressure-injected (Nanoliter 2000 injector, World Precision Instruments) into the brains at 2–3 different positions in the region of recording in order to reconstruct the position of the recording sites in standardized stereotaxic coordinates of a brain atlas of *P*. *discolor* (A. Nixdorf, T. Fenzl, B. Schwellnus, unpublished). The stereotaxic coordinates of these marker injections were documented and later compared to the positions of the marker in the stained tissue sections (Avidin-Biotin Complex / Diaminobenzidine staining procedure), which then served as references to the documented recording positions [20]. After the injection of the neuronal marker, the animals were euthanized by an intraperitoneally applied lethal dose of pentobarbital (0.16 mg/g bodyweight) and subsequently perfused transcardially.

### Characterization of basic delay tuning properties

In order to measure the basic echo delay tuning properties, the following procedure was used: a pre-recorded typical echolocation pulse of *P*. *discolor* containing no directional information [23] was presented with a constant level of 80–85 dB SPL. An “echo” was simulated by presenting the same pulse (without any spatial cues, no interaural differences) while randomly changing the echo delays from 1 to 29 ms (step size 3 ms) and the echo amplitudes from -45 to 0 dB (step size 5 dB) relative to the level of the “pulse”. Pulse and echo were presented diotically. The resulting neuronal activity was measured within a time window of 20–50 ms, starting directly after the echo presentation. Units’ responses were classified as “facilitated” if their response to at least one of the presented call/echo pairs was at least 30% stronger than the sum of the responses for only calls or only echoes, corresponding to [12]. As we wanted to investigate the spatial directionality of combination sensitive neurons, only units classified as “facilitated” were further analyzed with regard to basic echo delay tuning and spatial tuning characteristics.

To analyze the delay tuning of cortical units, delay response fields (DRFs) for facilitated units were visualized as filled contour plots, and threshold curves were calculated using a threshold of 50% of the maximum response for each unit. The DRF of each unit was further characterized by measuring the echo delay at the maximum response (best delay, BD) and the echo level at the maximum response (best level, BL), corresponding to Hechavarria et al. [24]. Cortical delay tuning maps were calculated by projecting the units’ positions in the AC onto the cortical surface [12].

### Characterization of three-dimensional tuning

**Fig 1:**
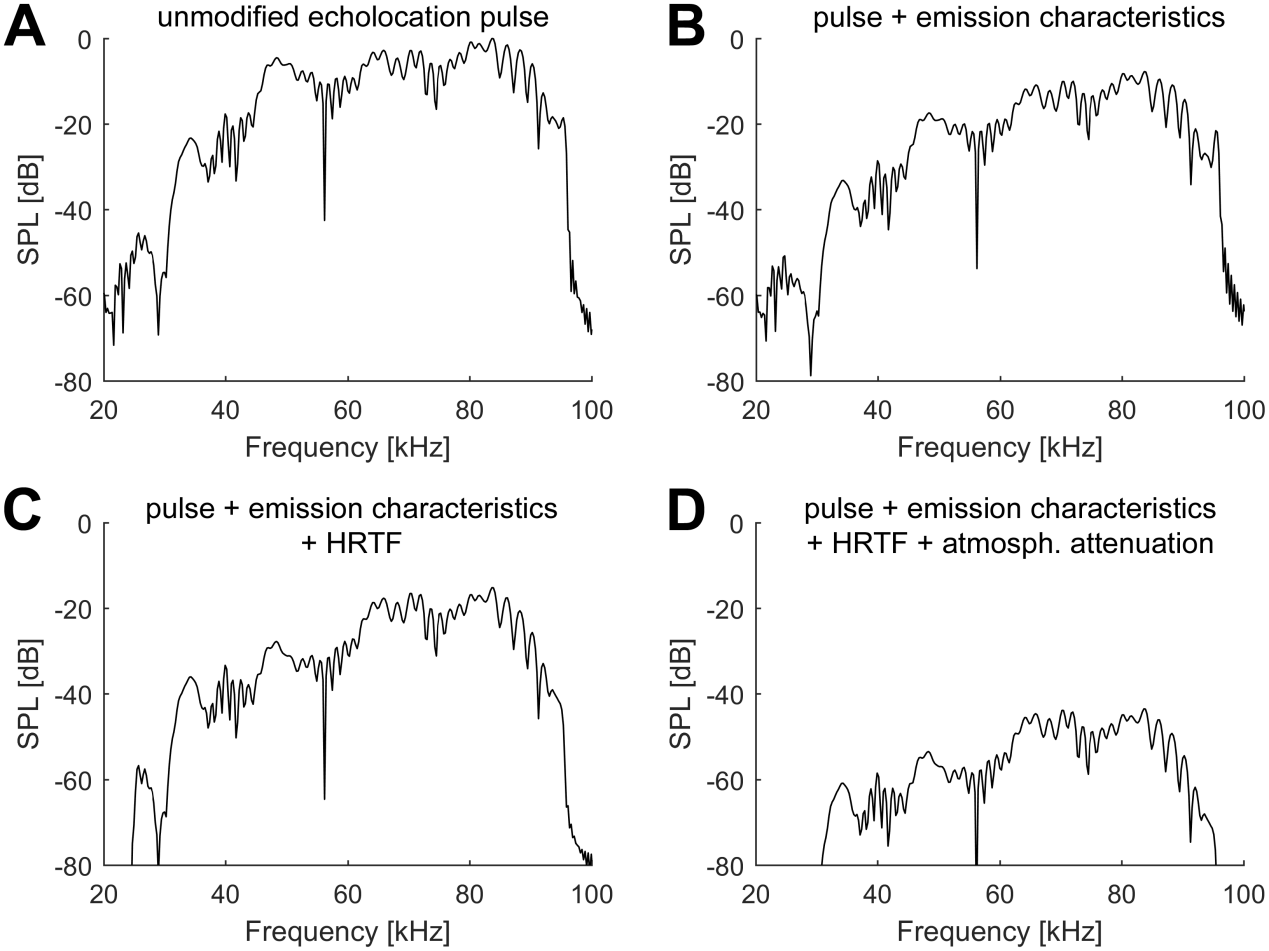
Frequency content of simulated echoes at different steps of generation. (A-D) Spectra of the simulated echoes (ipsilateral ear) derived from the original echolocation pulse at different steps of generation. The final echoes were simulated from a position of +22.5° azimuth and +7.5° elevation. The simulated target distance was 1.54 m, corresponding to an echo delay of 9 ms. For each panel, the x-axis shows the frequency and the y-axis the respective sound pressure level (SPL). (A) Spectrum of the unmodified, original echolocation pulse. (B) Spectrum of the echolocation pulse after simulation of the pulse emission characteristics. (C) Spectrum of the echoes derived from the original pulse and including pulse emission characteristics and HRTF. (D) Spectrum of the final echoes including pulse emission characteristics, HRTF and distance and frequency dependent atmospheric as well as geometric attenuation.

To test the spatial directionality of cortical delay tuned neurons, again a combination of artificial “pulse” and “echo” was used. The “pulse” was mimicked by using a prerecorded echolocation pulse of *P*. *discolor* containing no directionality. For the simulated “echo”, this pulse (see spectrum in Fig 1A) was convolved with the transfer function of call emission of *P*. *discolor* [25] (example in Fig 1B) and the left and right ear pair of a standardized, simulated head-related transfer function [26,27] (example in Fig 1C) corresponding to the tested spatial directions (from -67.5° to +67.5° in azimuth and elevation, spacing of 15.0°). We did not use individual HRTFs as the differences between the HRTFs of individual bats showed only a minor degree of inter-individual variability [26]. Geometric and atmospheric attenuation was calculated in Matlab. In detail, a vector of discrete frequency steps was generated ranging from 0 to 130 kHz in 10 kHz steps. A second vector contained the distance specific attenuations for each frequency step (derived from [28]), expressed in linear amplitude. In a next step, an impulse response was generated from both vectors using the Matlab fir2 function (signal processing toolbox). This impulse response was then convolved with the echoes to provide for frequency and distance specific atmospheric as well as geometric attenuation. Note, that atmospheric attenuation is the main determinant for echo attenuation at longer distances, while at shorter distance geometric attenuation is mostly determining echo attenuation. The resulting echoes included all relevant spatial cues, such as ITDs, frequency dependent ILDs and spectral profiles as well as distance-dependent and frequency-dependent sound attenuation (example in Fig 1D). Note that the presented echo level is always a function of the directional call emission level, the outer ear directionality and the traveled physical distance of pulses and echoes (corresponding to the echo delay). To mimic the typical level of self-stimulation by vocalization [29], the “pulses” were then further attenuated by 26 dB. Afterwards, “pulses” and “echoes” were combined including different tested echo delays.

Each unit’s spatial directionality was tested at an echo delay and absolute echo level corresponding to the unit’s best delay and best level as measured in the corresponding DRF. Additional records were performed at different shorter and longer echo delays for each unit. As described by Hagemann et al. [13] and due to our own experience in *P*. *discolor* [12], combination-sensitive neurons encoding different delay ranges are not uniformly distributed across the AC but show an overrepresentation of short echo delays. Furthermore, the delay-bandwidth of these neurons increases at longer echo delays [13]. Because of this phenomenon, we did not use linearly spaced but logarithmically space echo delay steps to investigate the spatial directionality of the combination sensitive neurons at different echo delays. The 5 standardized delay steps (X) were calculated using this formula:

$$echo\;delay=BD\times 1.25^{X}$$

X={−2, −1, 0, +1, +2}

Using this standardized, logarithmically spaced echo delay steps enabled us to directly compare each unit’s spatial directionality at different echo delays (corresponding to a physical distance to a virtual object) and calculate a three-dimensional receptive field for each tested unit. The presented call level was kept constant at different delay steps, the echo levels changed as a function of echo delay (i.e. distance dependent sound attenuation).

Further records were done at the units’ BD and echo levels of BL+10 dB and BL-10 dB to test the influence of the presented sound pressure level on the spatial directionality of the cortical delay tuned neurons.

For the analysis of the records, the neuronal activity was measured in the same time window as specified in the corresponding DRF (20–50 ms time window, starting directly after the echo presentation). Delay dependent spatial receptive fields (DSRF) were visualized as filled contour plots for each tested echo delay. The spike count in all records of each unit at different echo delays was normalized on the maximum response elicited in the recordings. In most units, the highest spike count was elicited at the units’ BD. Threshold curves were calculated for each record using a threshold of 50% of the normalized maximum response for each unit. The DSRFs were further characterized by measuring the spatial position eliciting the maximum response (best azimuth (BAZ), best elevation (BEL)), the centroid (geometric center in azimuth and elevation, Matlab function) and the width of the spatial receptive field (measured at the 50% contour line) for each delay step, corresponding to Suga et al. [15].

The results were statistically analyzed using a Kruskal-Wallis test for multiple comparisons, followed by a pair wise Bonferroni corrected Wilcoxon-Mann-Whitney test to investigate differences between distributions of different parameters. Correlation analysis was done using a Spearman’s rank correlation analysis (Matlab Statistics Toolbox). All parameters were considered as not normally distributed.

## Results

### Basic delay tuning properties

**Fig 2:**
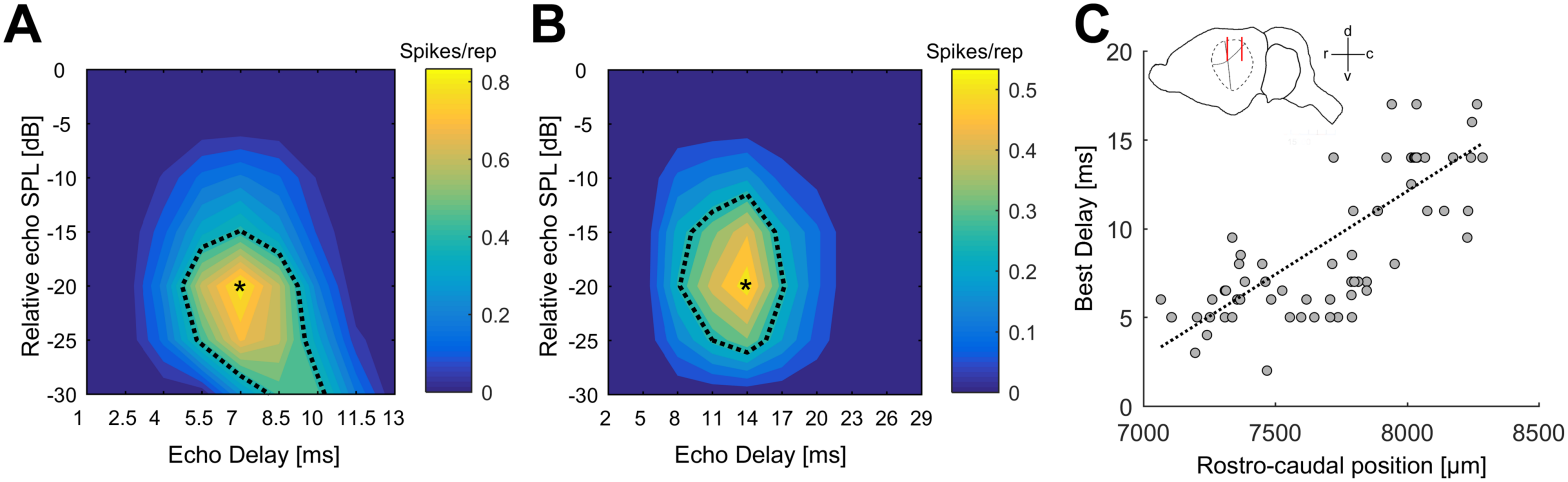
Delay tuning in the auditory cortex. (A,B) DRFs of a two cortical units with medium (A) and long (B) best delay. The spike count per repetition is color-coded; the black dotted contour line represents the response threshold at 50% of the units maximal spike count. The echo delay/echo level combination eliciting the maximal response is marked by a black asterisk (A: BD = 7 ms, BL = -20 dB, B: BD = 14 ms, BL = -20 dB). Note the different scaling of the abcissa in A and B. (C) Distribution of BDs along the rostro-caudal axis in the PDF of the AC. Units encoding short echo delays are located in the frontal part of the PDF, units with long best delays in the caudal part of the PDF. The dotted line indicates a linear regression. The position of the analyzed units in the cortex is sketched above the data points. Black lines indicate the bat’s brain, dashed lines indicate the position of the auditory cortex and red lines mark the rostro-caudal range from 7000–8500 μm as shown below. d: dorsal, v: ventral, r: rostral, c: caudal.

A total of 95 units were recorded in the posterior dorsal field (PDF) of the auditory cortex in 3 bats. 67/95 units (71%) showed a facilitated response to pairs of pulses and echoes. For all these units (n = 67), DRFs were obtained. Two examples are shown in Fig 2A and 2B. The unit in Fig 2A responds best at an echo delay of 7 ms (best delay = BD) at an echo level of -20 dB (best level = BL). The unit in Fig 2B responds at longer echo delays of about 14 ms at an echo level of -20 dB. The BDs for all 67 analyzed units ranged from 2.6 ms to 17.0 ms (median: 7.0 ms), the units’ BL ranged from -45 dB to 0 dB (median: -20 dB) relative to the presented pulse level (absolute echo level 40–85 dB SPL, median: 60 dB SPL). The BDs of the recorded units increased along the rostro-caudal axis (Fig 2C). Units with short BDs were significantly more rostral than those with longer BDs (RHO = 0.78, P < 0.001). No correlation could be found between the units’ BDs and the medio-lateral positions in the cortex (p > 0.05). BLs did not significantly correlate with the neurons’ positions in the auditory cortex.

### Spatial selectivity of neurons at best delay

**Fig 3:**
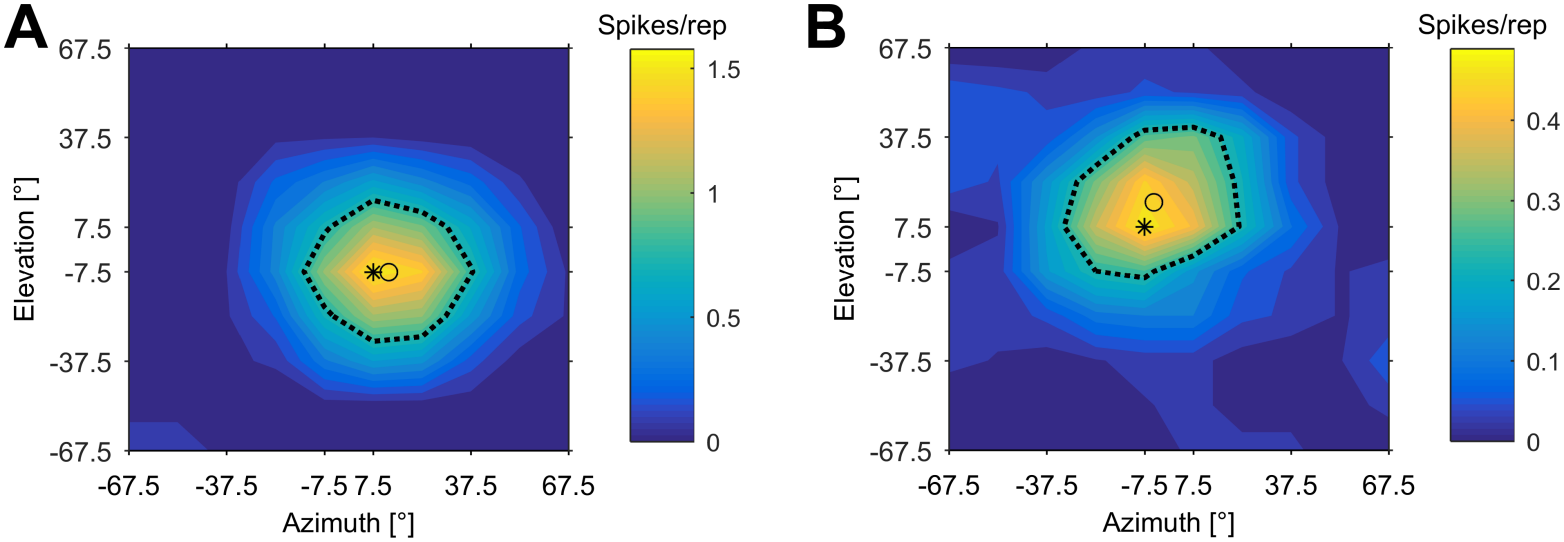
Spatial receptive fields at units’ BDs. DSRFs of two cortical unit’s with different best delays. The unit in (A) has a BD of 7 ms, the unit in (B) a BD of 14 ms. Pulses and echoes were separated by an echo delay corresponding to each unit’s BD. The spike count per repetition is color-coded. The spatial receptive field is represented by the black dotted contour line corresponding to the response threshold at 50% of each unit’s maximal spike count. The spatial position eliciting the maximal response is marked by a black asterisk; the geometric center of each unit’s spatial receptive field (centroid) is marked by a black circle. (A) This unit responded best at +7.5° azimuth and -7.5° elevation. The unit’s SRF centroid was at +12.9° azimuth and -6.9° elevation. (B) This unit responded best at -7.5° azimuth and +7.5° elevation. The unit’s SRF centroid was at -4.6° azimuth and +15.6° elevation.

For all units that showed a facilitated response to pairs of pulses and echoes (n = 67), spatial response characteristics were determined using pulse/echo pairs with an echo delay corresponding to each unit’s best delay and an absolute echo level corresponding to the each unit’s BL as measured in the DRF. Two examples are shown in Fig 3. The unit in Fig 3A (BD of 7 ms) responds best at a spatial position of 7.5° azimuth (best azimuth = BAZ) and -7.5° elevation (best elevation = BEL). The geometric center of this unit’s receptive field (centroid) is near the BAZ/BEL position at 12.9° azimuth and -6.9° elevation. The 50% contour line (dashed line in Fig 3) can be further used to measure the spatial tuning of the recorded neuron by determining the width of the receptive field in azimuth and elevation. The neuron shown in Fig 3A has a width in azimuth of 52° and a width in elevation of 48°. The unit in Fig 3B (BD of 14 ms) responds best at a spatial position of -7.5° azimuth and +7.5° elevation. The unit’s SRF centroid was at -4.6° azimuth and +15.6° elevation. The width of the receptive field was 54° in azimuth and 51° in elevation.

**Fig 4:**
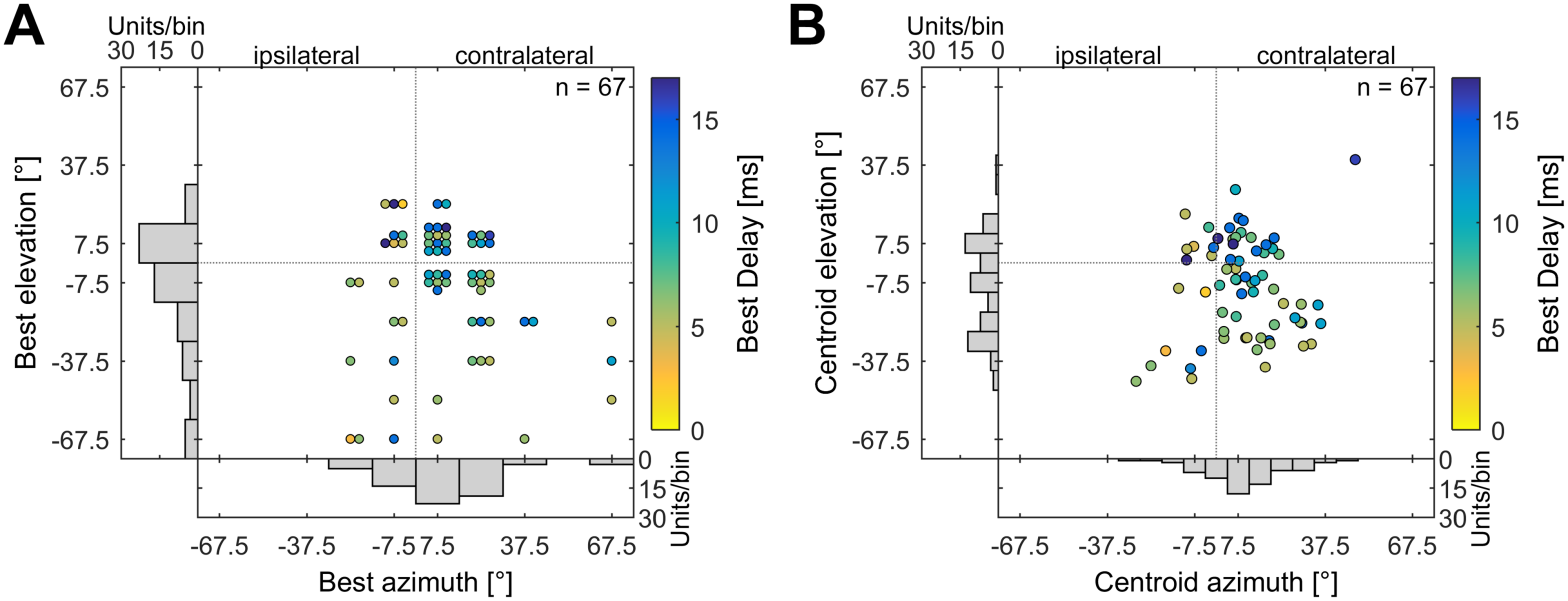
BAZ/BEL and centroid positions of all analyzed units. (A) Distribution of BAZ and BEL. The best responses were measured at discrete spatial positions (15° binning). Multiple units responding at the same position are grouped together in the figure. Each unit’s BD is color-coded. The lower histogram shows the distribution of BAZ and the left histogram the distribution of BEL. (B) Distribution of centroids of the spatial receptive fields. The lower histogram shows the distribution of the centroid positions in azimuth, the left histogram the distribution of the centroid positions in elevation (bin width 7.5°). All azimuth positions are normalized to one hemisphere. Azimuth positions > 0° correspond to positions contralateral to the recording site.

The BAZ/BEL positions for all analyzed units are shown in Fig 4A. The units’ BDs are color-coded. Most units respond best to echoes directly in front of the bat or in the lower contralateral quadrant. Note that due to the discrete positions recorded in azimuth and elevation, multiple units responded at the same spatial positions and are therefore grouped together in the figure. The BAZ ranged from -22.5° (ipsilateral) to 67.5° (contralateral), with a median at 7.5°. The BEL ranged from -67.5° (below the bat) to 22.5° (above the bat), with a median at -7.5°. As the BAZ/BEL were restricted to discrete positions in azimuth an elevation, we further analyzed the centroid positions of the spatial receptive fields to get a more detailed impression of the spatial directionality of these cells. The distribution of the centroids of all analyzed units is shown in Fig 4B and corresponds roughly to the BAZ/BEL positions. Most spatial receptive fields were targeted to frontal positions or to the lower contralateral quadrant. The centroid positions ranged from -27.6° to +47.9° (median: 8.5°) in azimuth and from -45.5° to 39.5° (median: -7.6°) in elevation.

**Fig 5:**
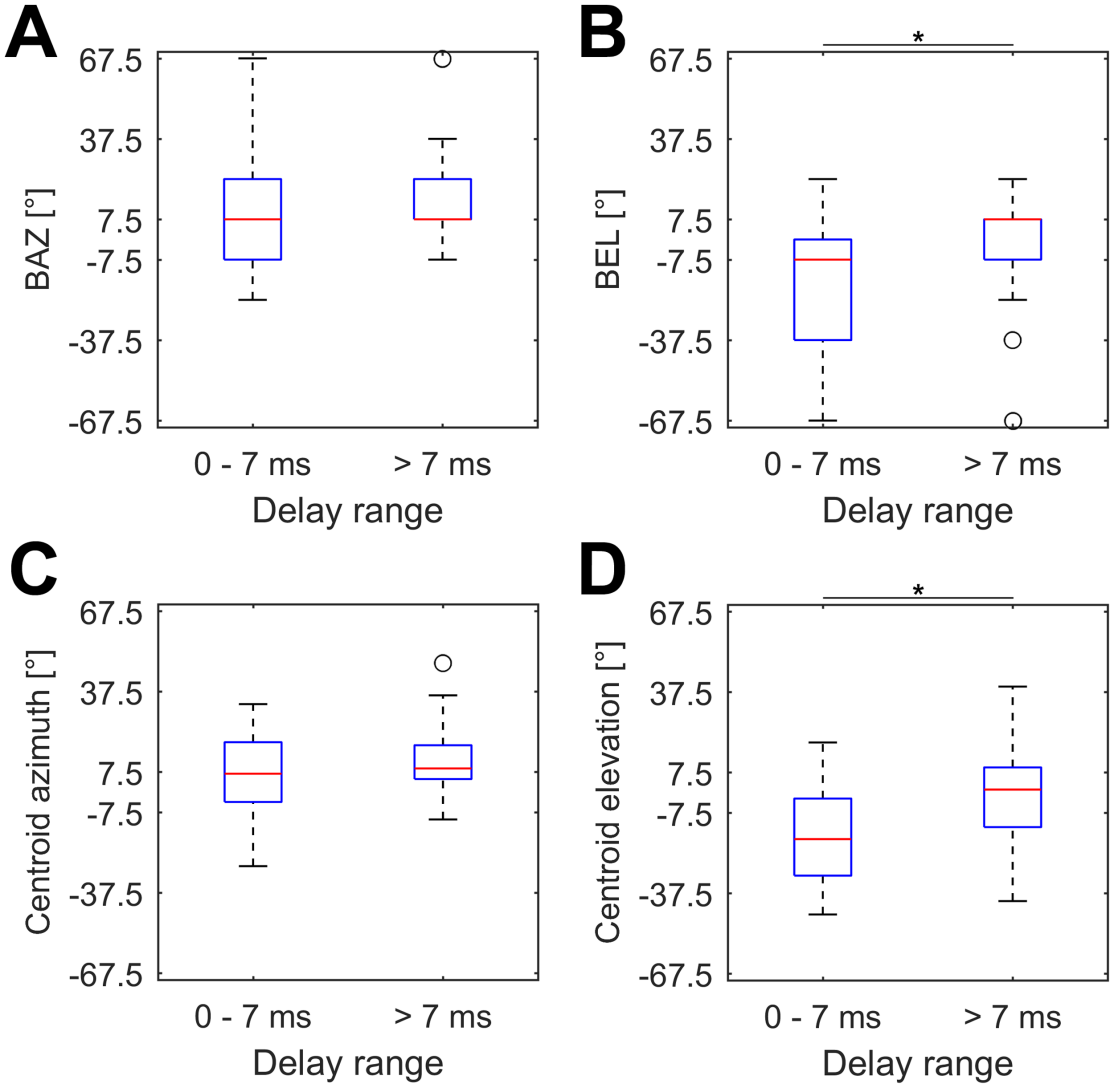
Azimuth and elevation at different best delays. Boxplots of BAZ (A), BEL (B), centroid positions in azimuth (C) and in elevation (D) for all analyzed units with best delays ≤ 7 ms (left box, n = 34) and > 7 ms (right box, n = 33). All boxes represent the median (red center line) and the interquartile range (IQR); whiskers indicate values within 1.5x IQR, black circles mark outliers. Significant differences between different groups are indicated by a black asterisk.

We further tested, if units with different best delays showed different spatial directionality. For this, units were separated in two equal sized groups according to their BDs. Units with short BDs (BD ≤ 7ms) tended to respond best to positions below the bat (median: -7.5, n = 34) while units with long BDs (BD > 7 ms) responded mainly to positions in the horizontal plane or slightly above (median: +7.5, n = 33). The differences of BEL between these two groups were significant (Wilcoxon: p < 0.01) and each unit’s BD and BEL were significantly correlated (Spearman: p < 0.01, rho = 0.33). However, the distribution of BAZ showed no differences between units tuned to shorter or longer echo delays (Fig 5A and 5B).

Corresponding to BAZ/BEL, the centroids from units with short BDs (BD ≤ 7ms, median elevation: -17.4°, n = 34) were located at lower elevations than centroids from units with long BDs (BD > 7ms, median elevation: +1.1°, n = 33). The differences between these two groups were significant (p < 0.01) and BD and centroid positions in elevation were again significantly correlated (Spearman: p < 0.01, rho = 0.37). No differences could be found for the centroid positions in azimuth with respect to different ranges of BD (Fig 5C and 5B).

We also checked a possible influence of BD and BL of all units on the size of their spatial receptive fields. We found a significant correlation between each unit’s BD and the width of the DSRF in azimuth (p = 0.01), but not between BD and the width in elevation (p > 0.05). DSRFs were significantly smaller in azimuth but not in elevation at longer BDs compared to short BDs. We found no correlation between each unit’s BL and the width of the spatial receptive fields in azimuth (p > 0.05) and elevation (p > 0.05).

We further investigated if the neurons spatial directionality depended on their location in the auditory cortex, or in other words, if delay tuned neurons form a topographic representation of space in the PDF of the auditory cortex. Our results show no correlation between each unit’s medio-lateral position in the cortex and the centroid position in azimuth (p = 0.14) or elevation (p = 0.35) and no correlation between each unit’s rostro-caudal position and centroid position in azimuth (p = 0.90). We found, however, a topographic arrangement of elevation along the rostro-caudal axis. Each unit’s centroid position in elevation and its rostro-caudal position were significantly correlated (p < 0.01). This means, neurons responding at short echo delays are positioned in the rostral part of the PDF and respond to lower elevations while neurons responding at longer echo delays are located in the caudal part of the PDF and respond preferably to positions in the horizontal plane or above.

We were wondering, if this correlation between BD and best elevation could be explained by the change of simple acoustic parameters due to our simulation and thus be just a simple epiphenomenon. Because of this, we investigated the influence of distance and frequency depended sound damping at different echo delays on the echo levels and echo frequency content. As the FM-sweeps of *P*. *discolor* contain most energy at around 60 kHz, we specifically analyzed echo levels at this frequency.

**Fig 6:**
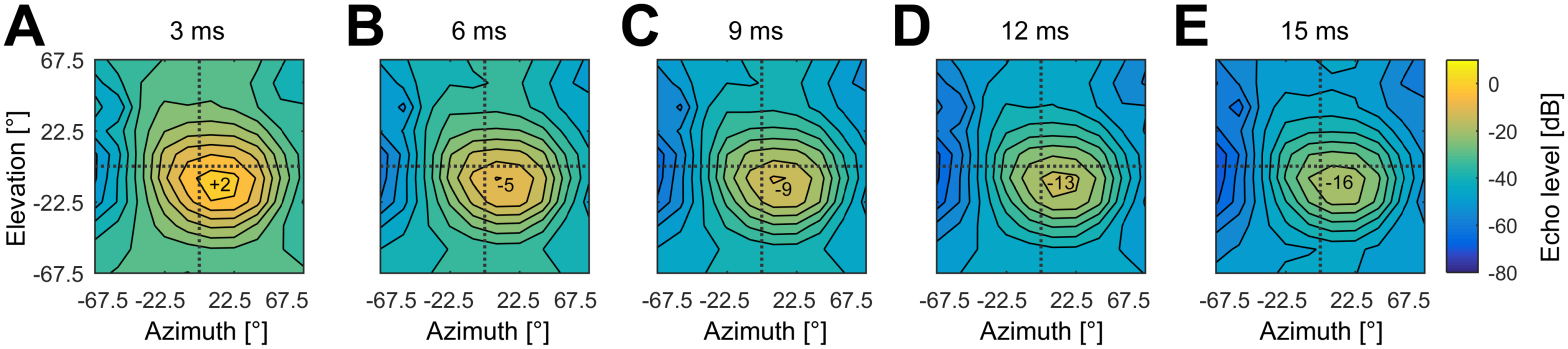
Simulated echo levels at different echo delays. Echo levels at 60 kHz relative to the presented pulse level at echo delays of 3 ms (A), 6 ms (B), 9 ms (C), 12 ms (D) and 15 ms (E). Relative echo levels are color-coded. The black lines correspond to differences of 5 dB. Echo levels at different spatial positions are calculated using the pulse emission characteristics of *P*. *discolor*, distance and frequency dependent sound damping (corresponding to the echo delay or physical distance in air) and the HRTF. For each echo delay, the maximum echo levels [dB] are indicated at the loudest position.

As shown in Fig 6, at an echo delay of 15 ms (≙ 2.6 m target distance), echo levels drop about 18 dB (at 60 kHz) compared to an echo delay of 3 ms (≙ 0.5 m). While the region of highest echo level remains quite stable at the same spatial position, the overall intensity increases. Thus spatial positions with low echo level in the 15 ms delay condition show higher echo levels in the 3 ms delay condition. If the maximum echo level in the 15 ms condition is near the neurons’ response threshold, the SRF should systematically increase in size and become more unspecific when echo delay decreases. However, this is clearly not the case in our data. As we will later on show (see analysis of three dimensional response properties), SRFs remain spatially focused even at short echo delays and SRFs of neurons with longer best delays shift to significantly higher positions in elevation but show no change in azimuth (compare Fig 5). Therefore, more complex neural processing must underlie the shape and size of SRF (e.g. additional neuronal computation of interaural level differences and/or monaural inhibitory interactions).

It would have been interesting to investigate the influence of the frequency content of the echoes on the spatial selectivity of the combination sensitive neurons in more detail. However, these neurons did not respond to presented pure tones. Because of this, it was not possible to gather basic data on frequency tuning characteristics. We further tried to use bandpass-filtered pairs of pulses and echoes, but found that again most units did not respond to these bandpass-filtered stimuli but only to broadband pairs of pulses and echoes.

We further analyzed the spatial tuning of the delay-tuned neurons by measuring the width of the spatial receptive fields of all units in azimuth and elevation at each unit’s BD and BL. The spatial receptive fields stretched across an angle of 45.7° up to 129.4° (median: 67.2°) in azimuth and 45.0° up to 118.9° (median: 64.8°) in elevation. As most units respond to frontal positions or positions in the lower contralateral space and span usually more than 45°, the receptive fields of these units show a high degree of overlap. Only the receptive fields of 9/67 (13%) units did not overlap with at least one other unit.

### Influence of call/echo level on spatial tuning

**Fig 7:**
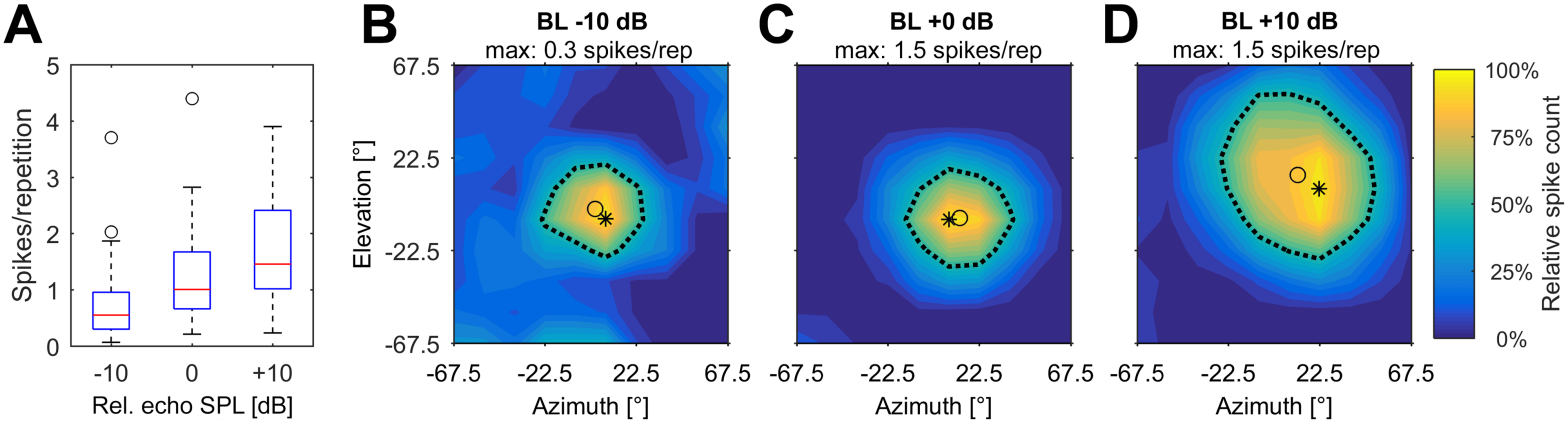
Influence of call/echo level on spatial tuning. (A) Boxplots of maximum spike count for all analyzed units (n = 40) and for each presented pulse/echo level. The boxes represent the median (red center line) and the interquartile range (IQR); whiskers indicate values within 1.5x IQR, black circles mark outliers. (B-D) Spatial receptive fields of a unit at the unit’s BD and echo levels of BL -10 dB (B), BL +0 dB (C) and BL +10 dB (D). The relative spike count in relation to the maximum response at each echo level is color-coded. The maximum spike count per repetition at each presented echo level is given on top of each figure. In each figure, the black asterisk marks the position of the maximum response and the black circle marks the centroid of the receptive field.

We further tested the influence of the presented pulse and echo level on the spatial directionality in a subset of the recorded neurons (n = 40). Note that, in our simulation, the echo level is always a function of the presented pulse level, echo delay and spatial position. Increasing pulse levels and consequently increasing echo levels led to considerably higher spike response rates in all units. At the spatial position eliciting the maximum response, the median spike count per repetition of all analyzed units was 0.6 at BL -10 dB, 1.0 at BL +0 dB and 1.5 at BL +10 dB (Fig 7A). Due to this increase of the spike count at increasing sound pressure levels, the width of each spatial receptive field was measured at the contour line corresponding to 50% of the unit’s maximum response at each presented echo level (BL -10 dB, BL +0 dB, BL +10 dB). An example of a unit responding at different presented call/echo levels is shown in Fig 7B–7D.

**Fig 8:**
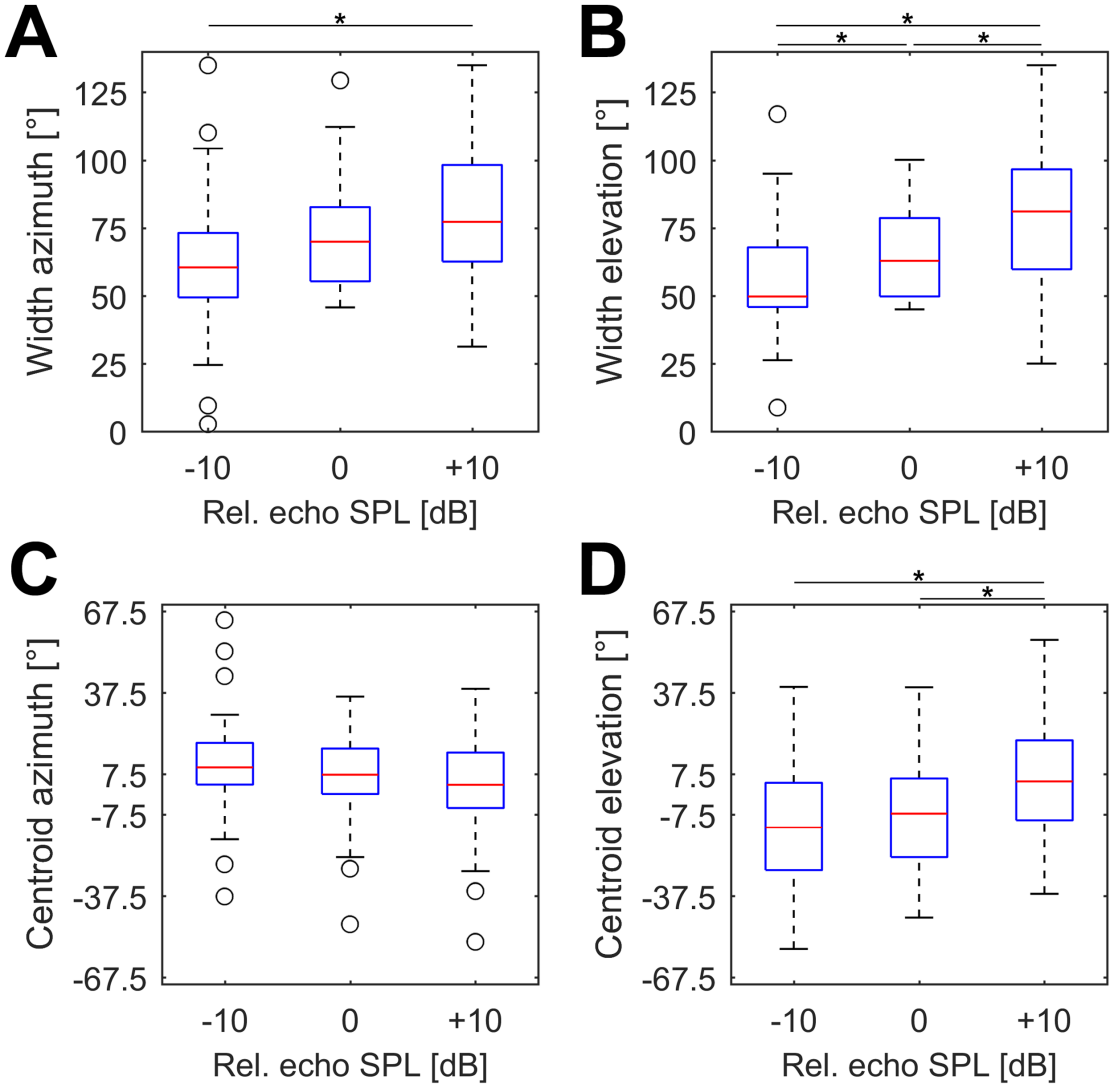
SRF width and centroid position of receptive fields at different echo levels. (A,B) Boxplots showing the widths of the spatial receptive fields of all analyzed units in azimuth (A) and elevation (B) at an echo level of BD -10 dB, BD +0 dB and BD +10 dB, respectively. (C,D) Boxplots showing centroid positions of the spatial receptive fields in azimuth (C) and elevation (D). Significant differences between different groups are indicated by a black asterisk (significance level after Bonferroni: 0.017).

The spatial receptive fields tended to broaden in azimuth and elevation at increasing sound pressure levels. The median width in azimuth was 60.5° (-10 dB), 69.9° (+0 dB) and 77.3° (+10 dB), respectively (Fig 8A). Differences in SRF azimuthal width were significant between relative echo levels of -10 dB and +10 dB (p = 0.003), but not between the other groups. The median width in elevation was 49.7° (-10 dB), 62.9° (+0 dB) and 81.1° (+10 dB), respectively (Fig 8B). SRF width was significantly different between an echo level of -10 dB and 0 dB (p = 0.015), 0 dB and +10 dB (p = 0.014) and -10 dB and +10 dB (p = 0.001). The Bonferroni corrected significance level (p value) for multiple testing was 0.017.

Furthermore, units tended to respond to more frontal position at higher sound pressure levels. The median azimuth centroid positions were 9.9° at -10 dB, 7.2° at +0 dB and 3.5° at +10 dB. However, the azimuth centroid positions were not significantly different between different pulse/echo levels (Fig 8C). The trend of responses to more frontal positions at higher pulse/echo levels was more pronounced for the centroid positions in elevation: the median elevation centroid positions were -12.3° at -10 dB, -1.2° at +0 dB and 4.8° at +10 dB (Fig 8D). Centroid positions in elevation differed significantly between a call/echo level of BD +0 dB and +10 dB (p = 0.016) as well as BD -10 dB and +10 dB (p = 0.006), but not between -10 dB and +0 dB (p = 0.67).

### Three dimensional response properties

**Fig 9:**
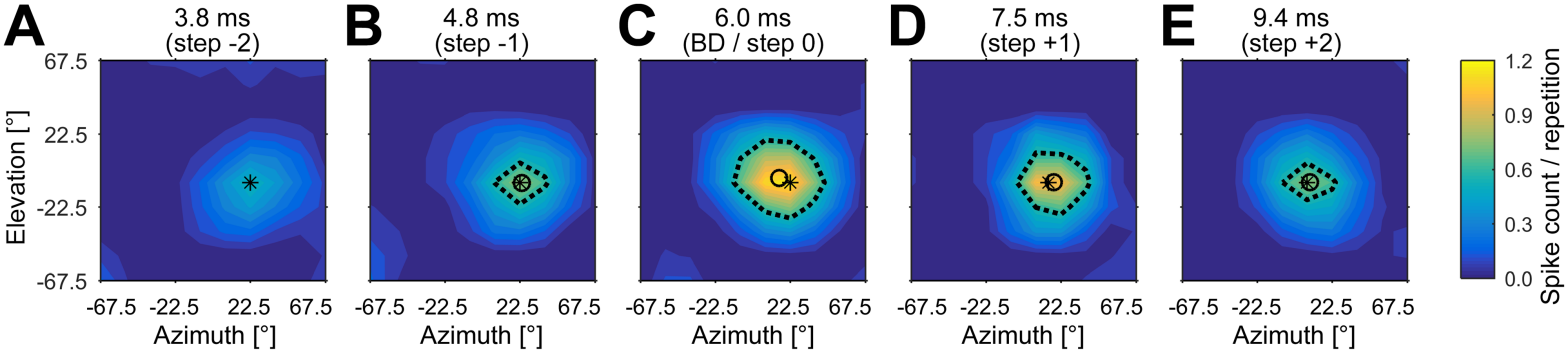
Spatial receptive fields at different echo delay steps. Example of a unit’s spatial receptive field at echo delay steps of 3.8 ms (A), 4.8 ms (B), BD = 6.0 ms (C), 7.5 ms (D) and 9.4 ms (E). The spike count per repetition is color-coded. All receptive fields at different echo delay steps are normalized on the maximum spike count evoked within these five delay steps. This unit responded best with 1.2 spikes per repetition at the unit’s BD of 6 ms. The maximum response position at each echo delay step is marked by a black asterisk; the receptive fields are indicated by a black dashed line (corresponding to 50% of the maximum response), and the centroid of each spatial receptive field is marked by a black circle. Note that, as the unit’s response drops below 50% of the maximum response rate at an echo delay of 3.8 ms, no spatial receptive field is shown.

In a subset of units (n = 27), we performed additional recordings at different standardized, logarithmically spaced echo delay steps (see Methods section), including each unit’s BD, to investigate the three dimensional response properties of the delay tuned neurons. An example for the response of a cortical unit to these five delay steps is shown in Fig 9. This unit shows its highest response rate at its Best Delay of 6.0 ms. The response to the presented stimuli decreases at shorter or longer echo delays. The spatial direction eliciting the maximum response, however, is in all recorded echo delay steps at approximate 22.5° in azimuth and -7.5° in elevation.

**Fig 10:**
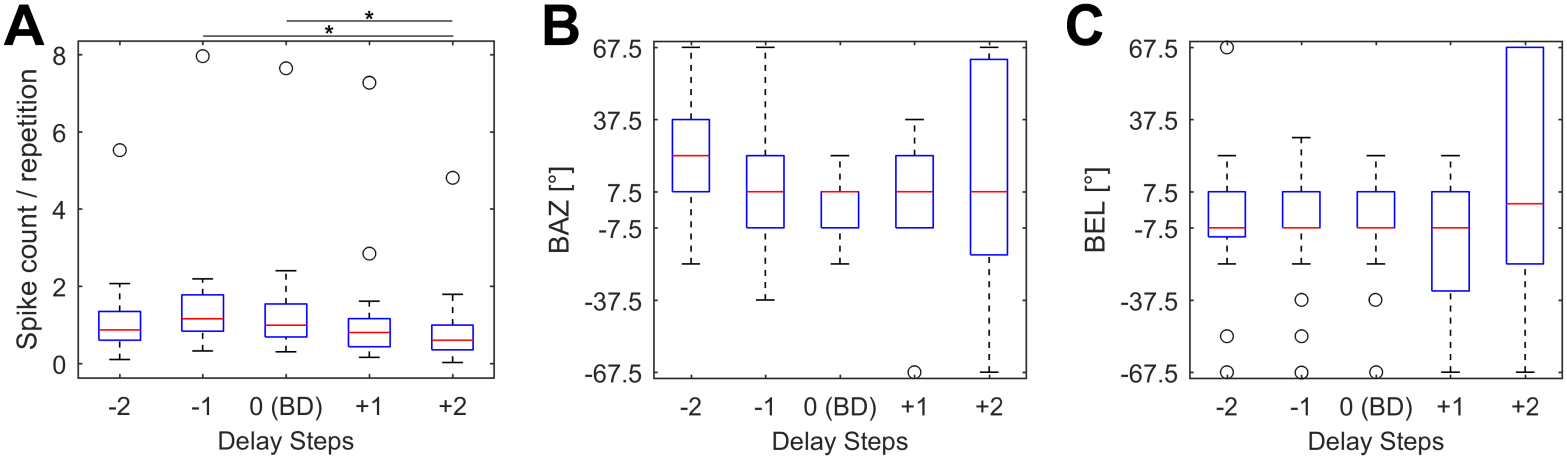
Maximum response and spatial directionality at different echo delay steps. (A) Boxplots of maximum spike counts per repetition for different, logarithmically spaced echo delay steps. The maximum number of spikes is elicited at delay step “-1” (one delay step shorter than each unit’s BD). Significant differences are indicated by a black asterisk. (B,C) Boxplots of BAZ (B) and of BEL (C) at different echo delay steps. No significant differences were found between different echo delay steps with respect to BAZ or BEL.

We analyzed the spike count and the spatial directionality of all 27 units at the different delay steps. Due to the DRFs of each unit, the highest spike count would be expected at each unit’s BD. In our data, however, the median maximum spike count was elicited at delay step “-1” (one delay step shorter than each unit’s BD, see Fig 10A). The median spike count per repetition was 0.87 (step -2), 1.16 (-1), 0.99 (0, corresponds to BD), 0.80 (+1) and 0.60 (+2). Significant differences between the spike counts were found only between steps -1 and +2 (p = 0.0007) and 0 = BD and +2 (p = 0.0099). The Bonferroni corrected significance level was 0.01. These results show that most units responded at a shorter echo delays when naturalistic “spatial” stimuli were presented in contrast to the measurements in the DRFs without any spatial information.

In some units, the neuronal response decreased below the threshold of 50% of the maximum response at the delay steps -2 or +2. Because of this, we could not analyze the centroid position for these neurons at these delay steps, but used BAZ and BEL as a measurement of spatial directionality at the different echo delays steps.

The analysis of BAZ and BEL for all 27 units clearly shows that the neurons respond to the same spatial direction at different echo delays (Fig 10B and 10C), independent of the changing echo levels due to distance dependent sound attenuation (see Fig 6). In contrast to the spatial directionality of units with different BDs, no significant differences were found between BAZ or BEL at different delay steps of the 3D fields. The median BAZ was 22.5° (step -2), 7.5° (-1), 7.5° (0 = BD), 7.5° (+1) and 7.5° (+2), respectively. The median BEL was -7.5° (step -2), -7.5° (-1), -7.5 (0 = BD), -7.5 (+1) and +2.5 (+2), respectively. In general, the variability of BAZ and BEL increases with increasing echo delays. An analysis of the centroid positions basically yields the same results (not shown here).

**Fig 11:**
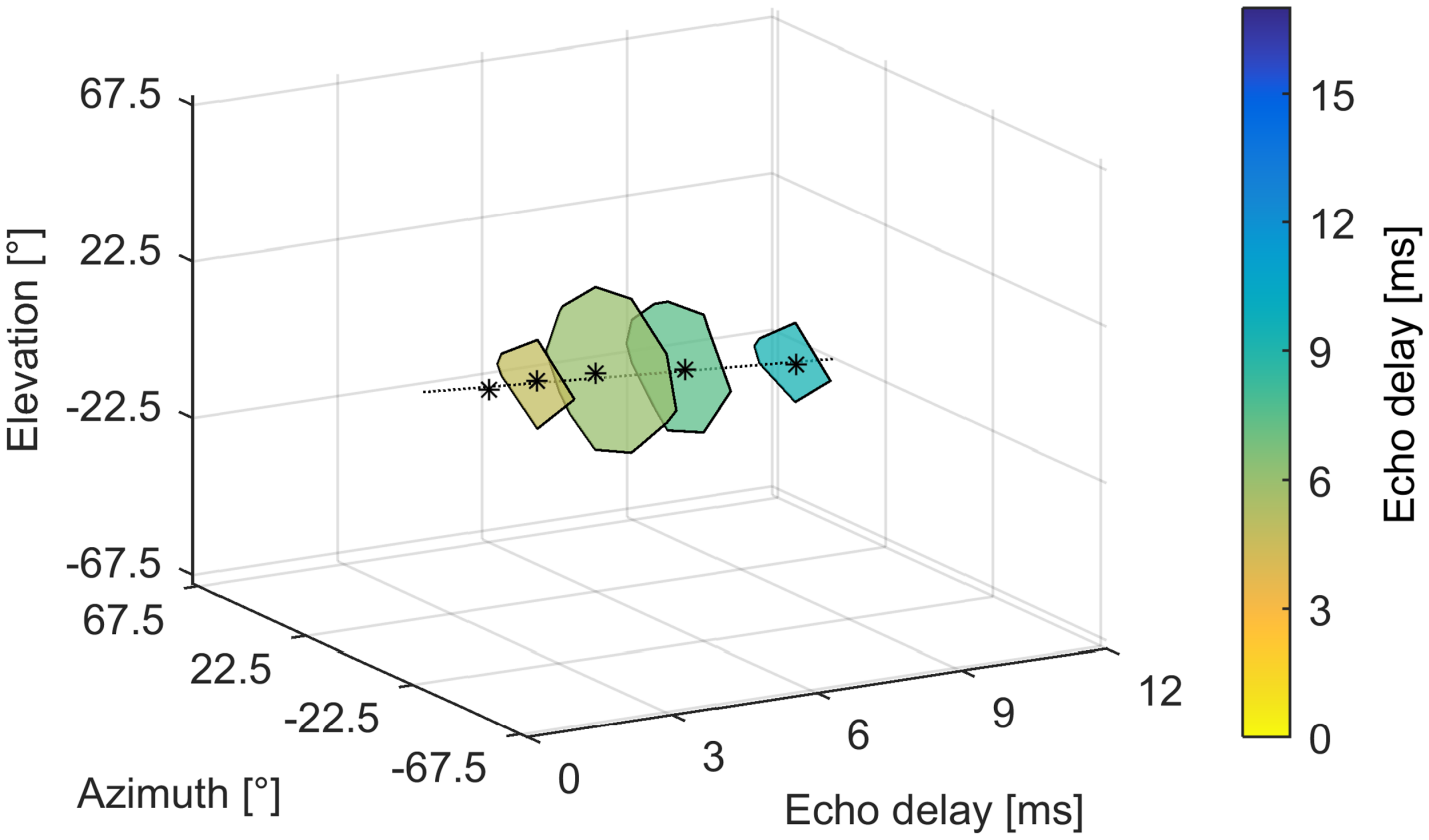
Three-dimensional receptive field of cortical combination sensitive neuron. Spatial receptive fields of the same unit as shown in Fig 9 in tree-dimensional space. The receptive fields (50% contour line) at each echo delay step (3.8 ms, 4.8 ms, 6.0 ms = BD, 7.5 ms, 9.4 ms) are indicated by a black solid line, the colored area indicates the respective echo delay and the black asterisks mark BAZ/BEL. The response rate and consequently the size of the receptive fields normalized on the maximum response at BD decrease at shorter or longer echo delays than the unit’s BD. Because of this, the black outlines of the receptive fields at different delay steps indicate a three-dimensional response volume in space where the response with at least 50% of its maximum response. The dotted black line shows the linear regression through BAZ/BEL at the different echo delay steps and indicates the unit’s spatial directionality.

Fig 11 shows an example of a three-dimensional receptive field. Due to the different spike counts at different echo delay steps, the unit exhibits an ellipsoid shaped, three-dimensional receptive field. The axis of the receptive field, through the BAZ/BEL positions at each echo delay step, gives an estimate of the unit’s directionality. The unit’s directionality is spatially constant across different echo delay steps.

**Fig 12:**
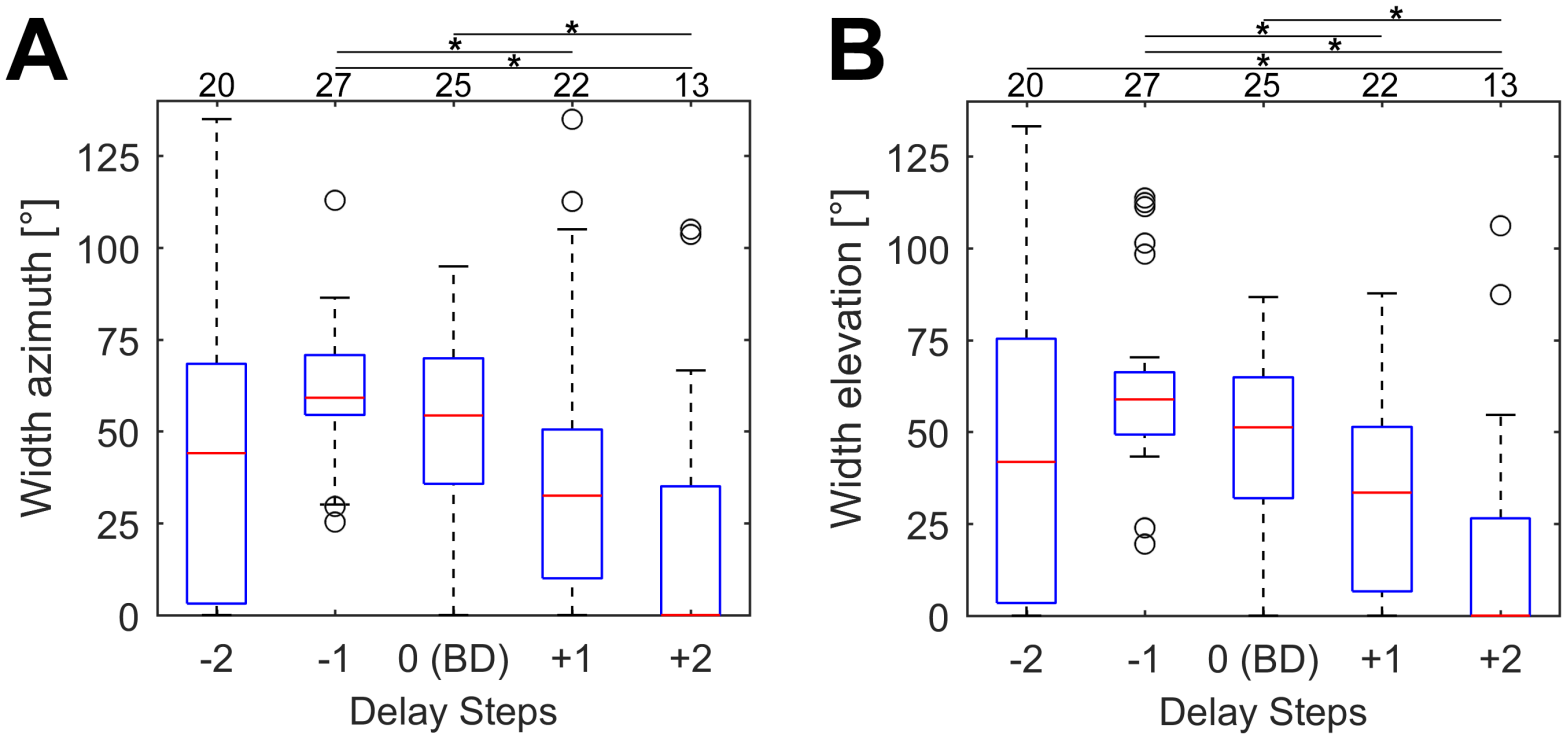
Sizes of the receptive fields at different echo delay steps. Boxplots showing width of the receptive fields in azimuth (A) and elevation (B) of all analyzed units (n = 27) at the logarithmically spaced echo delay steps. The unit’s show the broadest receptive fields at step -1. In part of the cells, the response drops below the threshold of 50% of the maximum response at some echo delay steps (no receptive field, width = 0°). The number of units exhibiting a receptive field width > 0° is noted above each figure. Significant differences between different delay steps in respect to field width in azimuth or elevation are indicated by a black asterisk.

The width of the receptive fields (measured as 50% contour line corresponding to each unit’s maximum response) at the different echo delay steps changed according to the spike count (Fig 12). The units exhibited the broadest receptive field at each unit’s delay step -1. At shorter or longer delays, the receptive field size decreased. In part of the cells, the response rate decreased below the threshold of 50% at some of the delay steps.

## Discussion

In the present study, we investigated the cortical representation of three-dimensional space in the echolocating bat *P*. *discolor*. We hypothesized that combination sensitive neurons encoding target distance can also process directional information. Our data reveal that neurons tuned to specific echo delays responded selectively to specific spatial directions, thereby mainly encoding positions in the horizontal plane and lower, contralateral space. We found a covariance of best elevation, best delay and rostro-caudal position of the combination-sensitive neurons in the AC. Neurons exhibited a higher spatial selectivity at lower sound pressure levels.

### Delay tuning and spatial selectivity

Our data on the delay tuning properties and the chronotopic distribution of the combination sensitive neurons in the PDF of the AC corresponds to recent studies in *Phyllostomus discolor* as well as closely related species [12,13]. Furthermore, this study clearly shows a selectivity of these combination sensitive neurons to specific spatial directions. The mean size of the spatial receptive fields in azimuth (~70°) and elevation (~63°) was well inside the range reported for cortical neurons in other bat species (about 60° in azimuth and 100° in elevation in pallid bats [4]) and considerably smaller than reported for other mammals (up to about 160° in humans [3], ≤140° in rhesus monkeys [30], ≤180° in cats [1,31,32]).

Interestingly, most neurons in our study encoded the frontal and lower contralateral space, while only few neurons encoded positions slightly above the horizon or on the ipsilateral side. When we combine the results from neurons in both hemispheres, these neurons encode a large spatial area to the front, below and to a lesser extend above the horizon. This region corresponds roughly to the biosonar receptive field shown for neurons in the superior colliculi of the midbrain in *P*. *discolor* [7]. Thus, the spatial selectivity of these neurons is focused on locations which might be most relevant for the echolocating bat: positions along the bats flight path and on the ground where prey can be expected. A comparable spatial selectivity of FM neurons for spatial positions along the flight path has already been described for pallid bats [4].

In contrast to Suga et al. [15] who proposed that directional information is not processed by the combination sensitive neurons, but is processed in parallel by a separate population of neurons, our data demonstrate that these neurons have well confined spatial receptive fields and are suited process directional information.

However, it is notable that the spatial selectivity depended on the presented sound pressure level. Neurons became less specific at higher sound pressure levels. Razak et al. [4] proposed, that the expansion of the spatial receptive fields with increasing sound levels might simply reflect ear directionality. The systematic enlargement of the receptive fields at increasing levels in our data fits well to this hypothesis.

The bats, however, might in some situations actually benefit from a lower spatial selectivity of these neurons as this facilitates target detection at specific distances. After target detection, an active control of sonar emission level, directionality and timing might help to optimize echo perception and target-clutter separation [33,34]. The targeting of ears and head might then enable more precise target localization by increasing spectral and intensity differences between the ears [35]. Furthermore, recent studies showed that dynamic stimulation leads to spatially more focused receptive fields and that echo-acoustic flow facilitates target segregation [12,36].

### Systematic representation of target elevation

**Fig 13:**
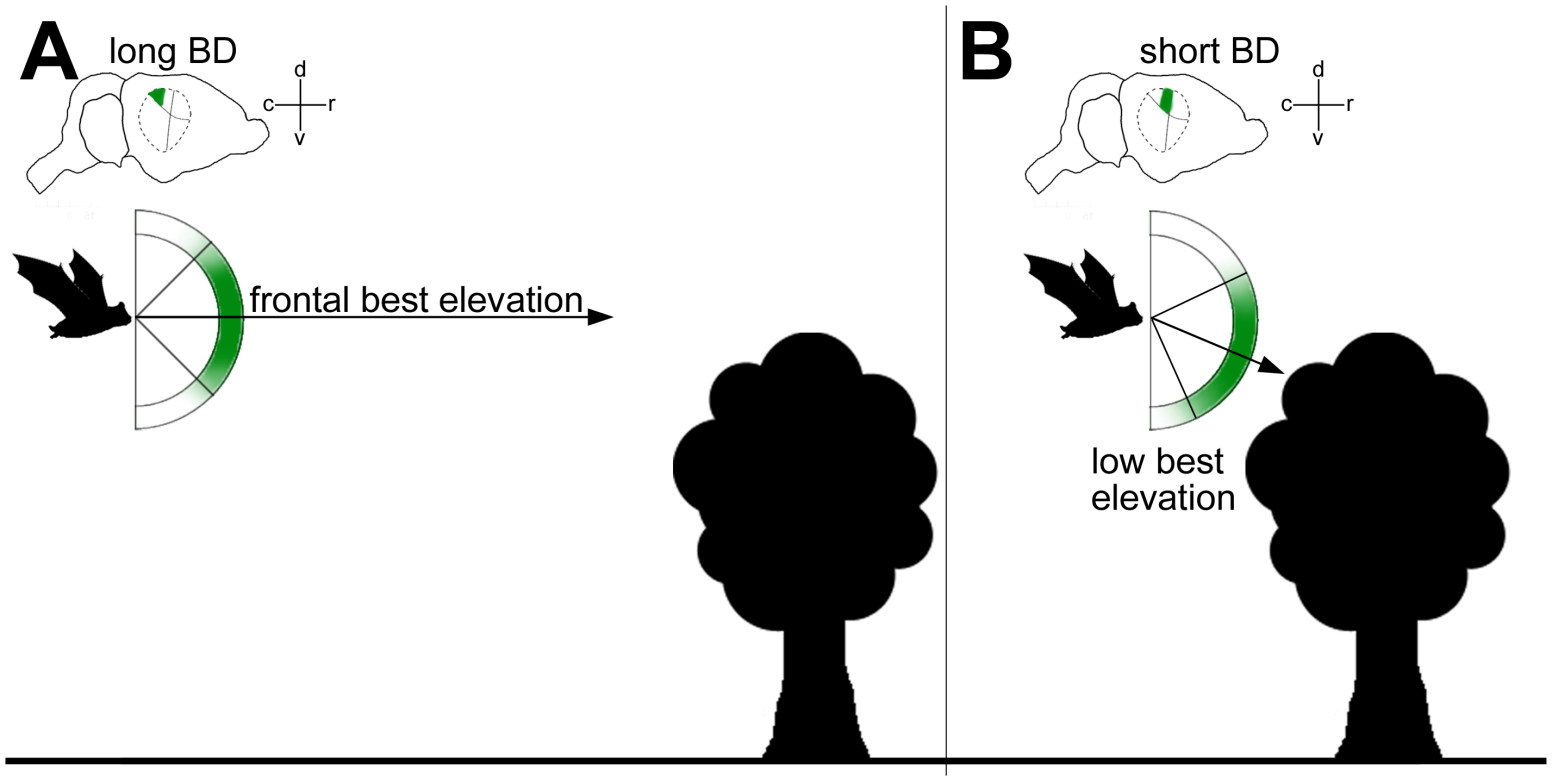
Systematic representation of target elevation and echo delay. Schematic drawing illustrating the possible implication of the covariation of BD and elevation for target representation in the cortical target-range map.. (A) At long distances to the target, neurons with long BDs in the caudal region of the PDF respond preferably to echoes from frontal positions. (B) At short distances to the target, neurons with short BDs in the rostral region of the PDF respond preferably to positions below the bat. Arrows represent the direction of best elevation. d: dorsal, v: ventral, r: rostral, c: caudal.

Our data show a covariance of best elevation, best delay and rostro-caudal position of the combination sensitive units in the PDF of the AC. This means, the representation of elevation is topographically aligned in the PDF. Interestingly, units tuned to long echo delays or distant targets responded to positions in the horizontal plane while units tuned to shorter echo delays or closer targets preferentially responded to positions below the horizon (Fig 13). This might be especially useful in a typical overfly situation encountered during foraging. During the approach to foraging grounds, the bats perception is targeted to distant objects along the flight path. At close range, however, when the bat is flying or hovering near a foraging tree, the perception is targeted more downwards towards possible food. Such an arrangement of best elevation might be further influenced by the bats typical foraging behavior: *P*. *discolor* is not an aerial hunter of insects, but forages around flowering trees and feeds on a mixture of nectar, pollen, fruits and insects among the trees [37]. Thus positions below the bat might be most relevant during foraging. At longer distances (e.g. during commuting flights) positions along a possible flight path might be more relevant.

We can further show, that this arrangement of best elevation cannot be explained by the change of single acoustic parameters like echo level as a function of echo delay. If the spatial directionality of these neurons would be mainly determined by the echo level and HRTF, one would expect more unspecific responses to the loud echoes at short echo delays. At longer echo delays and thereby more distance dependent sound attenuation, responses would become more specific to the most intense positions of the HRTF: near to horizontal plane and slightly contralateral. Our data, however, shows a high specificity of the neurons at all echo delays and a clear and consistent shift to lower elevations at short echo delays.

It seems most likely that, besides absolute echo levels, interaural level differences shape spatial directionality of the combination sensitive neurons, at least in azimuth. The influence of interaural level differences on azimuth selectivity in the auditory cortex was shown for different species, including bats [11,38,39]. However, a systematic investigation of the influence of interaural level differences on the spatial directionality of the combination sensitive neurons was beyond the scope of this study, but might be targeted in the future.

Hoffman et al. [16,17], who investigated the spatiotemporal response characteristics of cortical neurons, already found a spatial selectivity of combination sensitive cortical neurons in *P*. *discolor*. However, as they used no classical pulse/echo paradigm and did not simulate pulse emission characteristics and a change of echo levels due to the distance or echo delay, a direct comparison to the current the results is difficult. To our knowledge, no other study systematically investigated the topography of azimuth and elevation coding in the AC of *P*. *discolor* or closely related species so far. Unlike the topographic representations found in the visual cortex [40,41], the representation of acoustic spatial information in the auditory cortex in mammals remains a matter of debate [42,43]. While maps of auditory space are well established already on the level of the midbrain [44,45] a similar map has not been found in the auditory cortex of various mammals so far [46,47]. Neurons with similar binaural properties rather form local clusters than an orderly map [39]. This is surprising as numerous ablation studies have shown the importance of the auditory cortex (and different subfields within) for behaviorally measured sound localization [48,49]. A remedy for this might come from studies showing that spike timing and spike pattern might be more important than changes in firing rate (e.g. [50,51]). However, localization accuracy based on spike-timing of single neurons still proved to be insufficiently broad, putting forward the view that spatial position of sound sources are encoded via population codes based on either spike timing or firing rates [52,53].

The sound localization in bats, however, benefits from the high directionality of the sound emitting and sound receiving structures (i.e. nose leaf and pinnae) for frequencies in the ultrasonic range. This directionality might be reflected in the neural representation of space relatively i.e. in sharp spatial tuning of cortical SRF of *P*. *discolor* measured in our experiments.

Evidence for a special representation of auditory space in AC comes also from other bats. Razak [54] showed that in the primary tonotopic areas of the AC in pallid bats (*Anthrozous pallidus*), a systematic representation of sound azimuth exists. Importantly, this is not a point-by-point space map but space is encoded in form of a systematic change in the extent of activated cortex as azimuth changes from ipsilateral to contralateral locations. However, space representation in the auditory cortex of bats might differ between regions involved in passive hearing (e.g. when a bat listens to prey generated sounds) or regions involved in active echolocation. Razak et al. [4] showed that neurons in regions of the AC selective for echolocation specific frequency-modulated sweeps were more focused towards the midline while neurons in the noise-selective region were broadly tuned to contralateral azimuth. SRF size and target elevation were correlated with the characteristic frequency for neurons in the noise selective region but not in the frequency-modulated sweep-selective region in these bats. Our results reveal now for the first time a topographic-like representation of elevation of the combination-sensitive neurons in the PDF of the AC in *P*. *discolor*.

### Three-dimensional representation of space

The systematic recordings at different echo delay steps revealed a clear tuning of the combination sensitive neurons to three-dimensional space. Interestingly, most units responded best at an echo delay shorter than each unit’s best delay (derived from the DRF). This might be influenced by the higher echo levels at shorter echo delay steps in our simulation or simply by the limited precision we used to determine the basic delay tuning properties of each unit in the corresponding DRF. However, there might also be a difference between the echo delay tuning of cells when measured with pulse/echo combinations containing spatial information (e.g. interaural level differences and spectral differences) in contrast to the measurements without any spatial cues as in the DRFs. Studies using naturalistic flight sequences to determine the response characteristics of combination sensitive neurons already presented evidence, that a dynamic change of spatial and temporal information has a significant impact on the delay tuning properties of these neurons [12,23].

However, our data clearly show that each unit has a “specific” delay, corresponding to a physical distance to the target, and a “specific” spatial direction in azimuth and elevation where it responds best. Interestingly, neurons exhibited the same spatial directionality at all presented echo delay steps. Despite the fact that the echo levels and echo frequency content change according to the different echo delay steps (as a function of distance and frequency depended sound damping), neurons spatial directionality remained constant for each neuron at different delay steps. This means that each neuron specifically encodes a direction and a specific delay range, or in other words, a specific volume in three-dimensional space (see Fig 11).

At a non-optimal echo delay, the spike response and consequently the size of the spatial receptive fields decrease. Because of this, the neurons responses become more “selective” to specific spatial directions. This indicates that neurons near to, but not exactly at their best delay might be better suited for target localization. The same effect of a higher spatial selectivity could be shown at lower pulse/echo levels when tested at each unit’s best delay. This means that by actively adjusting pulse/echo level during target approach, the bat can increase its target localization abilities. It is important to note that the bat can not only increase or decrease its vocalization level, but can of course adjust multiple biosonar parameters like sonar beam width, direction and frequency content as well as temporal parameters to optimize sensory acquisition [19,55].

Moreover, it is most likely that an active orienting behavior including targeting of head and ears at specific targets enables a more flexible and on the same time precise localization. As mentioned above, these combination sensitive neurons most probably do not form a static representation of three-dimensional space. According to the dynamic nature of the target distance map [12,23] and the reported sharpening of spatial receptive fields evoked by dynamic stimulation [36], it seems most likely that the three-dimensional response properties of these cortical combination sensitive neurons are further modified by echo-acoustic flow information and active orienting behaviors like sonar vocalization pattern or head and pinna movements as well as top-down attentional effects [35,56].

### Conclusion

We found, that combination-sensitive neurons in the AC of *Phyllostomus discolor* can process target distance information as well as directional information and are thereby suited to encode a three-dimensional representation of space. Our data further reveal a topographic distribution of best elevation i.e., neurons in the rostral part of the target distance map representing short delays prefer elevations below the horizon. Top-down attentional effects and echo-acoustic flow information in natural scenes might further help to increase spatial selectivity and allow are more precise target discrimination.
